# Resting and exercise-induced occult hypertension and coronary atherosclerosis in male masters endurance athletes

**DOI:** 10.1136/bjsports-2025-111347

**Published:** 2026-04-24

**Authors:** Jamie O’Driscoll, Gemma Parry-Williams, Joanna Moser, Ioannis Vlahos, Daniel Obaid, Joyee Basu, Gherardo Finocchiaro, Saad Fyyaz, Hamish MacLachlan, Sarandeep Marwaha, Chris Miles, Efstathios Papatheodorou, Nicola Gaibazzi, Domenico Tuttolomondo, Damini Dey, Maximiliano Moreira, Athanasios Bakalakos, Thijs Eijsvogels, Vincent Aengevaeren, Aneil Malhotra, Maria Tome-Esteban, Rajan Sharma, Michael Papadakis, Sanjay Sharma

**Affiliations:** 1 Diabetes Research Centre, College of Life Sciences, University of Leicester, Leicester, UK; 2 Leicester Diabetes Centre, Leicester General Hospital, University Hospitals of Leicester NHS Trust, Leicester, UK; 3 St George’s University Hospitals NHS Foundation Trust, London, UK; 4 Cardiovascular and Genomic Research Institute, City St George’s University of London, London, UK; 5 Cardiovascular Clinical Academic Group, St George’s University of London, London, UK; 6 Department of Radiology, St George’s University Hospitals NHS Foundation Trust, London, UK; 7 University of Texas MD Anderson Cancer Centre Brain Tumor Centre, Houston, Texas, USA; 8 Institute of Life Sciences, Swansea University Medical School, Swansea, UK; 9 Cardiologia Centro, Azienda Ospedaliero-Universitaria di Parma, Parma, Emilia-Romagna, Italy; 10 Biomedical Imaging Research Institute, Cedars-Sinai, Los Angeles, California, USA; 11 Department of Medical BioSciences, Exercise Physiology Research Group, Radboud University Medical Centre, Nijmegen, The Netherlands; 12 Department of Cardiology, Radboud University Medical Centre, Nijmegen, The Netherlands; 13 Institute of Sport, Manchester Metropolitan University, Manchester, UK; 14 Heart, Vascular and Thoracic Institute, Cleveland Clinic London, London, UK

**Keywords:** Athletes, Cardiology, Exercise

## Abstract

**Objective:**

Recent studies have demonstrated a greater prevalence of coronary atherosclerosis in male masters endurance athletes, but the underlying contributors remain unclear. We explored the relationship between occult resting and exercise-induced hypertension with coronary atherosclerosis characteristics.

**Methods:**

198 male masters endurance athletes with a low Framingham risk score (<10%) and no clinical diagnosis of hypertension underwent 24-hour ambulatory blood pressure (ABP) monitoring and exercise BP assessment. Coronary CT angiography assessed coronary artery calcification (CAC) score, luminal stenosis and high-risk plaque features.

**Results:**

Seventy-eight (39%) athletes were hypertensive on ABP monitoring and 93 (47%) demonstrated a hypertensive response to exercise. A CAC score of 1–99 Agatston units (AU), 100–399 AU and ≥400 AU was present in 94 (47%), 32 (16%) and 15 (8%) athletes, respectively. Twenty-four (12%) athletes had coronary stenoses >50%. Sixty-two athletes (31%) had calcified plaque, 32 (16%) had mixed plaque, 2 (1%) had non-calcified plaque and 26 (13%) had markers of high-risk plaque. Hypertension on ABP monitoring was significantly associated with a CAC score ≥100 AU (OR: 2.56; 1.08 to 6.04) and coronary stenosis >50% (OR: 2.92; 1.17 to 7.33). A hypertensive response to exercise was significantly associated with coronary stenosis >50% (OR: 4.72; 1.65 to 13.5) and the presence of high-risk plaque (OR: 3.27; 1.27 to 8.43).

**Conclusion:**

Masters male endurance athletes have a high prevalence of occult hypertension, which is associated with high-risk features of coronary atherosclerosis. Both ambulatory and exercise-induced hypertension are associated with a higher prevalence of atherosclerotic coronary artery disease in male endurance athletes. Early identification and timely clinical management of this classic cardiovascular disease risk factor may reduce the burden of coronary atherosclerosis in athletes.

**WHAT IS ALREADY KNOWN ON THIS TOPIC**

**WHAT THIS STUDY ADDS**

- Both ambulatory and exercise-induced hypertension are independently associated with adverse coronary plaque phenotypes, including higher CAC burden, luminal stenosis and high-risk plaque morphology. This study demonstrates that traditional risk factors, such as abnormal ambulatory and exercise blood pressure responses, are associated with adverse coronary phenotypes in masters athletes.

**HOW THIS STUDY MIGHT AFFECT RESEARCH, PRACTICE OR POLICY**

- Early detection of occult hypertension in masters athletes provides an opportunity for targeted risk modification and preventive interventions, potentially reducing the burden of subclinical coronary atherosclerosis. These results may inform future athlete screening guidelines, encouraging a more nuanced approach to blood pressure evaluation in older endurance athletes.

## Introduction

 Regular engagement in exercise training is associated with a lower risk of adverse cardiovascular events.^1^ However, male masters endurance athletes with seemingly low cardiovascular disease risk have shown an increased prevalence of high coronary artery calcium (CAC) scores and a greater number of coronary plaques compared with relatively sedentary counterparts of similar age.^2–5^ Elevated CAC, significant stenoses, and high-risk morphology plaques are strongly associated with increased risk for major adverse coronary events.^6^ Importantly, middle-aged males account for more than 90% of all exercise-related sudden cardiac deaths,^7^ with atherosclerotic coronary artery disease (CAD) being responsible for over 80% of cases.^8 9^

The cause of accelerated atherosclerotic CAD in male masters endurance athletes remains unknown;^2 10^ however, classic cardiovascular disease risk factors appear to be significant contributors.^11^ Hypertension is the leading modifiable risk factor for cardiovascular disease, playing a central role in the development and progression of CAD in the general population.^12^ The relationship between hypertension and CAD has not been previously explored in lifelong athletes with a seemingly low Framingham risk score. Therefore, we assessed the relationship between hypertension (24-hour ambulatory and exercise-induced) with CAC score, coronary artery plaque, and high-risk plaque features in male masters endurance athletes.

## Methods

### Study population

**Figure 1:**
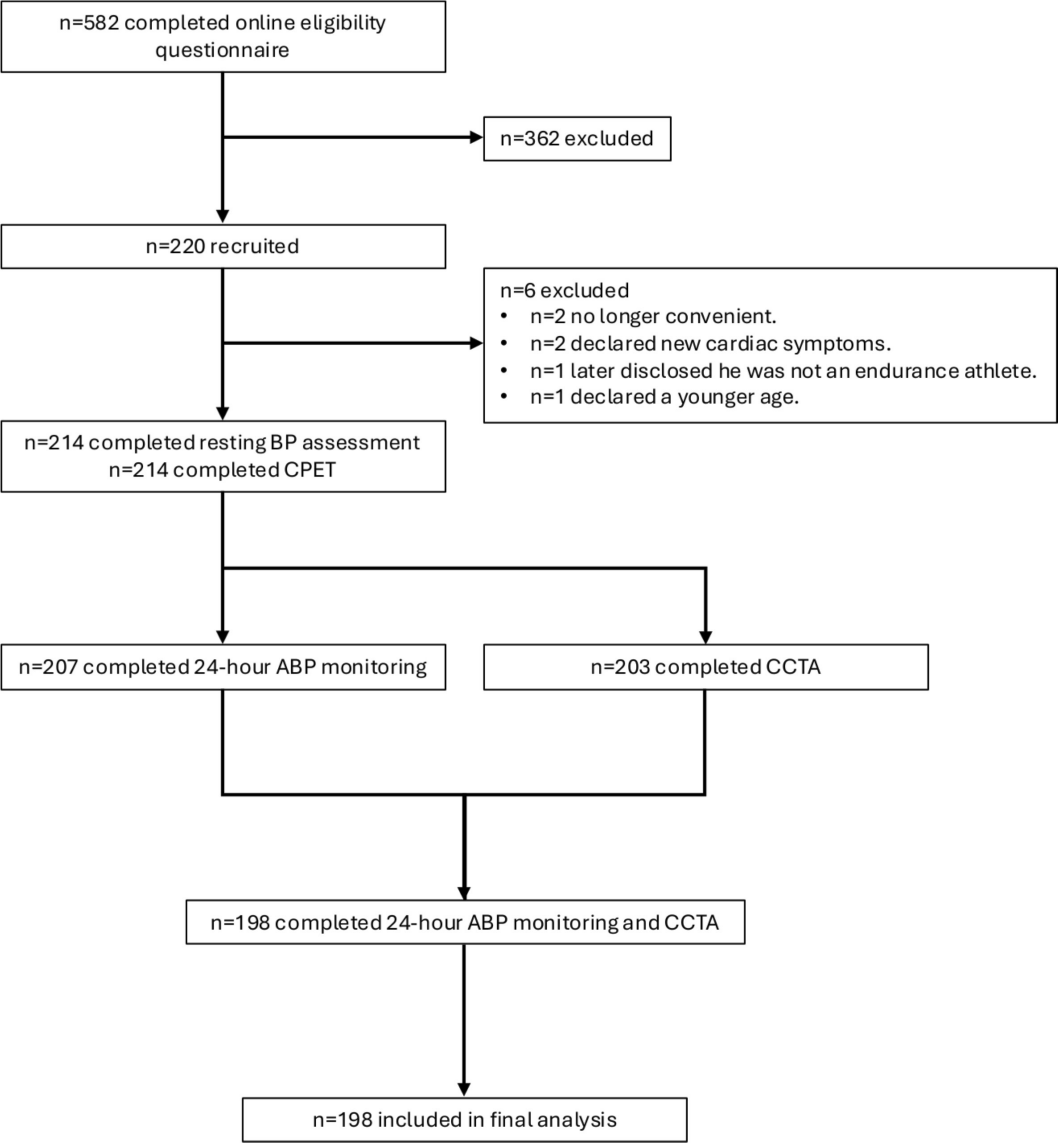
Study flow diagram. Study flow diagram illustrating the number of participants assessed for eligibility, excluded participants, participants who completed all study procedures and those included in the final analysis. ABP, ambulatory blood pressure; BP, blood pressure; CPET, cardiopulmonary exercise test; CCTA, coronary CT angiography.

Between February 2018 and March 2019, a total of 220 male masters endurance athletes were recruited (figure 1). Following exclusions, an analytical cohort comprising 198 athletes underwent physical examination, symptom-limited cycle ergometer cardiopulmonary exercise testing (CPET), 24-hour ambulatory BP (ABP) monitoring, CT coronary calcium scoring and coronary CT angiography (CCTA).

Athletes were recruited from running, cycling and triathlon clubs throughout the UK. Eligibility criteria included males aged 40–65 years who participated in endurance exercise for a minimum of 6 hours per week. Exclusion criteria included current cardiac symptoms including chest pain, shortness of breath, palpitations, cardiogenic syncope or presyncope; a recent deterioration in exercise capacity, previous history of cardiac disease, active or former smoking history, known hypertension, diabetes mellitus, severe hypercholesterolaemia (low-density lipoprotein cholesterol >4.9 mmol/L), chronic kidney disease (estimated glomerular filtration rate (eGFR) <90 ml^−1^⋅min^−1^⋅1.73 m^2^) or any other chronic systemic or inflammatory conditions, a Framingham risk score >10%, any contraindication to CT, and refusal of consent to sharing of abnormal results with the primary care physician.

### Online eligibility questionnaire

Eligibility for the study was determined by an online questionnaire, which enquired about basic demographics, presence or absence of cardiac symptoms, risk factors for atherosclerosis, and exercise history. A more detailed exercise questionnaire was used to elicit information about the principal sporting discipline(s), competitions and personal bests, the average hours per week of each sport, number of days of exercise per week, number of months of training intensively in a year, total number of years of endurance training and the time spent exercising at a specific intensity. The estimated exercise dose was acquired from the questionnaire and calculated in metabolic equivalent of task units (METs) based on activity type by applying guidance from the International compendium of physical activities.^13^ Lifelong MET hours were calculated by multiplying METs/week × hours per week × number of months of endurance training per year × total number of years of endurance training.

### Physical examination

Physical examination included height (cm), weight (kg), and resting clinic ‘office’ BP. Resting clinic BP was measured and defined according to current European Society of Cardiology guidelines.^14^ Individuals with hypertension detected during these measurements were not excluded if their calculated Framingham risk score was sufficiently low. Athletes also underwent biomarker assessment (venous blood sample) to identify and exclude diabetes mellitus and hypercholesterolaemia, as well as to calculate their Framingham risk score.

### Cardiac investigations

#### Cardiopulmonary exercise testing

CPET was performed in an upright position with a COSMED E100W cycle ergometer (Rome, Italy) using a ramp protocol of 25–30 Watts/min according to the athlete’s exercise history and height. Participants were encouraged to exercise to maximal volitional exhaustion. Breath-by-breath gas exchange analysis was performed using a dedicated COSMED Quark CPEX metabolic cart (Rome, Italy). Predicted peak aerobic capacity was calculated using age, sex, height and weight-adjusted reference equations.^15^ Predicted maximal heart rate was calculated using the Astrand equation (220 - age). BP was recorded manually by a single operator at intervals of 2 min throughout the test and for 6 min of recovery. Peak oxygen consumption (p$\overset{˙}{\text{V}}$O_2_) was used to determine the current level of physical fitness, and a peak systolic BP ≥210 mm Hg was used to define a hypertensive response to exercise.^16^

#### 24-hour ambulatory blood pressure monitoring

A 24-hour ABP monitor (ABPM 1700, Welch Allyn, NYC, USA) was attached, and athletes were encouraged to continue their usual day-to-day life activities, excluding exercise or anything requiring vigorous movement during the investigation. Measurements were made using an automated system with a protocol of hourly measurements from 06 hours to 09 hours and 21 hours to 01 hour and measurements every 20 min between 09 hours to 21 hours and 01 hour to 06 hours, to obtain sufficient data for interpretation according to the European Society of Cardiology/European Society of Hypertension guidelines,^14^ while minimising discomfort and inconvenience to the athlete. Hypertension was defined according to a 24-hour ABP ≥130/80 mm Hg, daytime ABP ≥135/85 mm Hg, or night-time ABP ≥120/70 mm Hg according to current European Society of Cardiology guidelines.^14^

#### Coronary CT angiography (CCTA) and calcium scoring

CCTA was performed using a wide-area detector 320-slice Revolution GE scanner (GE Healthcare) and prospective gating using a commercially available protocol (SnapShot Pulse, GE Healthcare), capable of single rotation full cardiac coverage. The estimated total radiation dose was 10 mSv. The following scanning parameters were used: a scout scan was performed, followed by a prospectively gated calcium score scan (gantry rotation time of 270 ms, 120 kVp and ~124 mA) with 16 cm z-axis coverage from the tracheal bifurcation to the diaphragm (field of view, 25 cm). CCTA was acquired with prospective gating and a 0.63×0.63 mm resolution, using a medium-soft tissue convolution kernel (standard), and individual size-adapted kVp, mAs, and iterative reconstruction settings to achieve minimum diagnostic dosimetry. No athlete required intravenous Metoprolol as all had a heart rate of <60 beats per minute. Participants received two 400 mcg doses of sublingual Glyceryl trinitrate. For the angiographic part of the scan, 100 mL of iohexol (Omnipaque 350 mg I/ml) contrast, at a flow rate of 5 mL⋅s^−1^ followed by 100 mL of saline solution, was injected into an antecubital vein via an 18-gauge peripheral venous catheter. Bolus tracking was used with a region of interest placed in the ascending aorta. Image acquisition was then commenced once signal intensity reached a predefined threshold of 100 Hounsfield units. The CAC score was plotted according to percentiles for age and luminal stenosis, and plaque morphology was visually assessed using established guidelines.^17 18^

All CT scans were co-reported by two expert Consultant Radiologists using a standard clinical reporting system, including CAC score, stenoses (a significant stenosis considered >50% in a major vessel), plaque morphology (ie, calcified, non-calcified or mixed) and plaque vulnerability markers (ie, spotty calcification, ruptured plaque, positive remodelling, low attenuation plaque and napkin ring sign,^19^ that measures the necrotic core to fibrous cap ratio).

### Primary and secondary analysis

The primary variable for analysis was CAC score ≥100 Agatston unit (AU). Secondary measures were a CAC 1–99 AU, CAC 100–399, CAC >400 AU, stenosis ≥50% in any coronary artery, coronary plaque morphology (calcified, mixed and non-calcified) and the presence of high-risk plaque vulnerability markers (spotty calcification, ruptured plaque, positive remodelling, low attenuation plaque and napkin ring sign).

### Power analysis

This prospective observational study was powered based on the assumption that the prevalence of significant CAC ≥100 AU in this population is approximately 18.8%.^2 4^ The primary explanatory variable was hypertension, with age and lifetime exercise dose included as additional covariates. To ensure a robust multiple logistic regression model, 10–16 outcome events per predictor variable are recommended. With three explanatory variables, this corresponds to a required 30–48 cases of significant CAC. Assuming a CAC ≥100 prevalence of approximately 20%, a total sample size of 200 participants (minimum of 188 participants) would yield the necessary number of events. To allow for up to 10% missing data, the final target sample size was set at 220 participants.

### Equity, diversity and inclusion statement

The author team consists of men and women from diverse professional backgrounds, seniority levels, countries and disciplines. Our study population consisted of male masters athletes from a broad geographical and socioeconomic range.

### Statistical analysis

Statistical analyses were performed using SPSS Statistics V.29 (IBM New York, USA). All variables were assessed for normality both visually using normality plots and the Shapiro-Wilk test. Skewness and kurtosis values were also examined to evaluate the distribution of continuous variables. Continuous variables were reported using mean±SD, or median (IQR) as appropriate. Differences between athletes with and without hypertension on ABP monitoring, as well as those with and without a hypertensive response to exercise, were assessed using either the independent samples t-test or the Mann–Whitney U test, depending on the distribution of the data. Categorical data were reported as proportions, and differences between groups were assessed using the χ^2^ test. For our primary analysis, binary logistic regression was used to calculate unadjusted and adjusted ORs for the association between hypertension (24-hour ABP monitoring and exercise-induced) and CAC >100 AU. We decided a priori to adjust for the following confounding factors: age, lifetime exercise dose and resting clinic BP. These statistical procedures were repeated for our secondary outcomes (CAC >0, CAC >400, coronary stenosis >50% and presence of high-risk plaque features). An alpha level of 0.05 was set as the threshold for statistical significance.

## Results

### Baseline demographics

A total of 198 (90%) athletes of the original 220 recruited completed all study procedures (figure 1). Athletes were aged 51±7 years, mostly white (94%) and had a Framingham risk score of 3.9±2.6%. Over half of the cohort (62%) were triathletes, and the remainder were endurance runners (23%) and cyclists (16%). Athletes exercised for 9.3 (7–12) hours per week, with a cumulative exposure to endurance exercise of 16 (9–30) years and a lifetime exercise dose of 17 963 (8478–37 572) MET-hours.

### Clinic and ambulatory blood pressure profile

**Figure 2:**
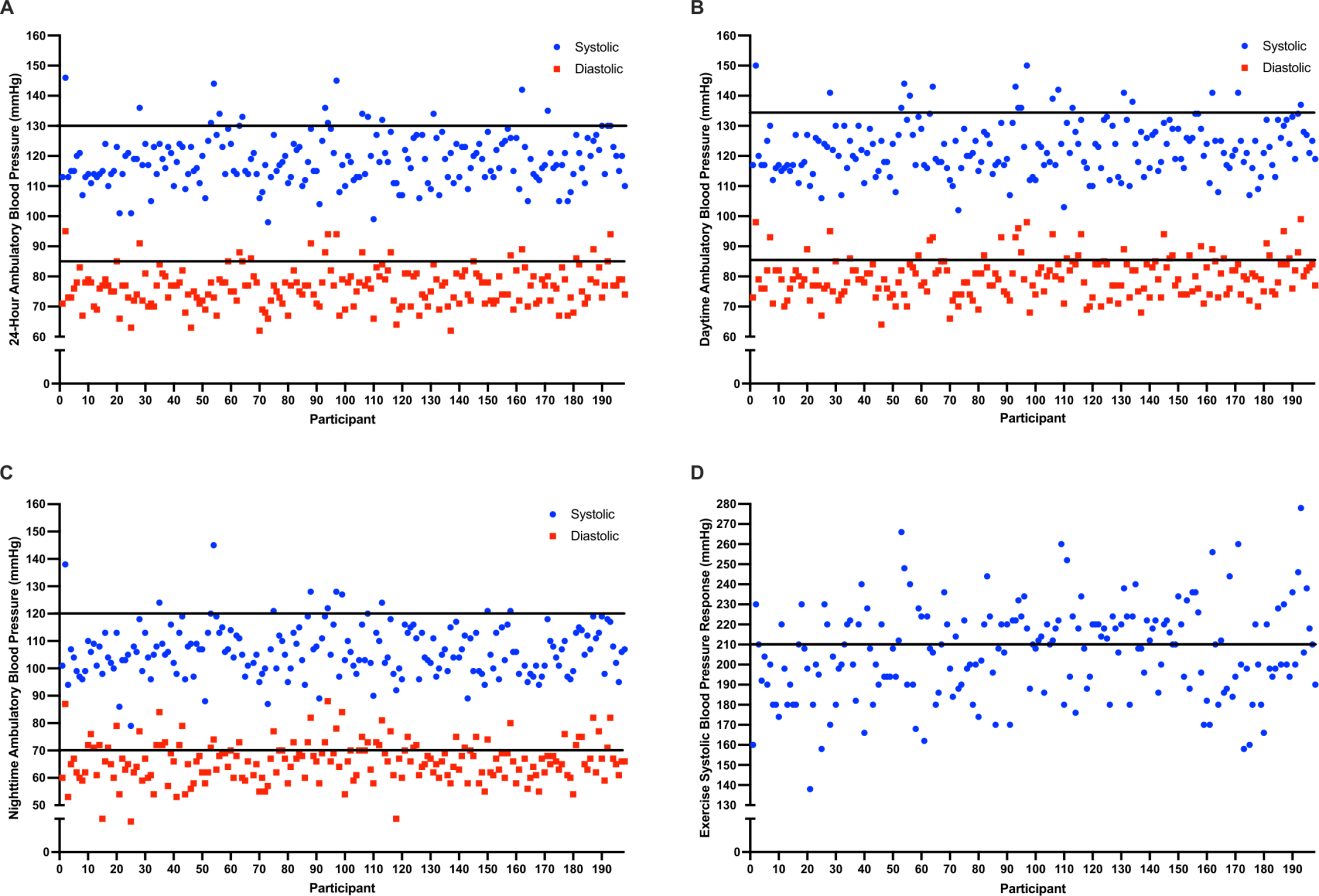
Individual participant level data. (**A**) 24-hour systolic and diastolic ambulatory blood pressure (ABP), (**B**) daytime systolic and diastolic ABP, (**C**) nighttime systolic and diastolic ABP and (D) peak exercise systolic blood pressure, with threshold lines indicating the diagnostic criteria for hypertension (A, B and C) and a hypertensive response to exercise (**D**).

Resting clinic BP was measured in all participants. The mean clinic systolic and diastolic BP was 129±14.8 mm Hg and 80±8.2 mm Hg, respectively. Individual participant data for ABP outcomes are presented in figure 2. The average 24-hour systolic and diastolic ABP was 118±11 mm Hg and 76±6 mm Hg, respectively, with a daytime mean ABP of 123±9 mm Hg and 80±7 mm Hg and nighttime mean ABP of 106±10 mm Hg and 66±7 mm Hg. In total, 78 (39%) participants were hypertensive on ABP monitoring.^14^ Of these, 57 (73%) met 24-hour ABP criteria (≥130/80 mm Hg), 45 (58%) met daytime ABP criteria (≥135/85 mm Hg) and 55 (71%) met nighttime ABP criteria (≥120/70 mm Hg). Notably, 70 (90%) of these athletes had a normal clinic BP, indicating a prevalence of occult hypertension that would not have been identified by resting clinic measurements alone.

There were no significant differences in age, distribution of sporting disciplines, cardiovascular fitness, or lipid profile between normotensive and hypertensive athletes (table 1). However, hypertensive athletes had a significantly greater peak exercise systolic and diastolic BP, as well as a substantially greater proportion eliciting a hypertensive response to exercise compared with normotensive athletes. In addition, hypertensive athletes had a slightly higher fasting blood glucose level compared with normotensive athletes; however, none had levels compatible with impaired glucose tolerance or diabetes mellitus (table 1).

**Table 1:** Baseline demographics, exercise performance and cardiovascular risk factors in normotensive and hypertensive athletes, and in athletes with normotensive or hypertensive responses to exercise

Characteristics	Normotensive (n=120)	Hypertensive (n=78)	P value	Normotensive blood pressure response (n=105)	Hypertensive blood pressure response (n=93)	P value
Demographics						
Age (years)	51.3±6.5	51.6±7.7	0.777	50.8±7.0	52.2±7.1	0.161
Height (cm)	178.8±6.7	177.4±7.0	0.084	178.2±6.2	178.2±7.6	0.990
Weight (kg)	74.6±9.2	75.9±8.0	0.287	74.0±8.0	76.3±9.3	0.063
White ethnicity (n, %)	112 (93.3)	74 (94.9)	0.658	98 (93.3)	88 (94.6)	0.704
Exercise performance						
Triathlon (n, %)	74 (61.7)	48 (61.5)	0.986	62 (59)	60 (64.5)	0.430
Running (n, %)	29 (24.2)	16 (20.5)	0.549	28 (26.7)	17 (18.3)	0.160
Cycling (n, %)	17 (14.2)	14 (17.9)	0.474	15 (14.3)	16 (17.2)	0.573
Duration of endurance exercise training (years)	20.6±13.1	20.1±14.5	0.808	18.9±12.5	22.2±14.7	0.081
MET hours per week	113.3±84.7	116.8±62.7	0.769	113.5±88.6	116.0±60.8	0.652
Peak $\dot{V}$O_2_ (ml⋅kg^−1^⋅min^−1^)	50.1±7.2	49.5±6.6	0.548	49.7±6.7	50.1±7.3	0.648
Per cent predicted $\dot{V}$O_2_ (%)	154.3±20.5	154.6±17.2	0.921	151.8±19.7	157.4±18.3	0.042
Peak work rate (W)	330.5±53.6	332.7±51.2	0.774	325.6±50.3	337.9±54.5	0.101
Peak METs	14.3±2.1	14.1±1.9	0.543	14.2±1.9	14.3±2.1	0.650
Peak heart rate (b⋅min^−1^)	159.2±24.1	162.2±22.8	0.388	160.8±20.4	159.8±26.8	0.773
Peak systolic blood pressure (mm Hg)	200.4±22.0	215.0±21.4	<0.001	189.0±13.9	225.5±13.5	<0.001
Peak diastolic blood pressure (mm Hg)	76.6±8.3	80.4±8.8	0.002	76.9±8.1	79.4±9.2	0.039
Hypertensive response to exercise (n, %)	41 (34.2)	52 (66.7)	<0.001	–	–	–
Cardiovascular risk						
24-hour systolic ABPM (mm Hg)	114.1±11.4	124.4±7.9	<0.001	113.8±12.2	123.0±7.8	<0.001
24-hour diastolic ABPM (mm Hg)	72.9±4.2	81.6±5.3	<0.001	74.3±5.8	78.7±6.1	<0.001
Daytime systolic ABPM (mm Hg)	119.0±6.9	128.3±8.9	<0.001	118.6±7.3	127.3±8.5	<0.001
Daytime diastolic ABPM (mm Hg)	76.4±4.5	84.8±6.4	<0.001	77.5±6.1	82.1±6.6	<0.001
Nighttime systolic ABPM (mm Hg)	102.3±7.9	112.7±8.9	<0.001	102.8±8.6	110.3±9.5	<0.001
Nighttime diastolic ABPM (mm Hg)	62.2±4.9	71.9±6.4	<0.001	64.0±6.4	68.2±7.6	<0.001
Total cholesterol (mmol⋅L^−1^)	5.1±0.9	5.1±0.8	0.724	5.1±0.8	5.1±0.9	0.813
LDL cholesterol (mmol⋅L^−1^)	2.7±0.8	2.8±0.7	0.752	2.8±0.8	2.7±0.8	0.952
HDL cholesterol (mmol⋅L^−1^)	1.8±0.5	1.8±0.4	0.405	1.8±0.4	1.8±0.5	0.848
Blood glucose (mmol⋅L^−1^)	4.8±0.8	5.1±0.9	0.005	4.9±0.9	5.0±0.7	0.408
Framingham risk score (%)	3.7±2.5	4.2±2.7	0.162	3.5±2.4	4.3±2.8	0.031

ABPM, ambulatory blood pressure monitor; HDL, high-density lipoprotein; LDL, low-density lipoprotein; MET, metabolic equivalent of task.

**Figure 3:**
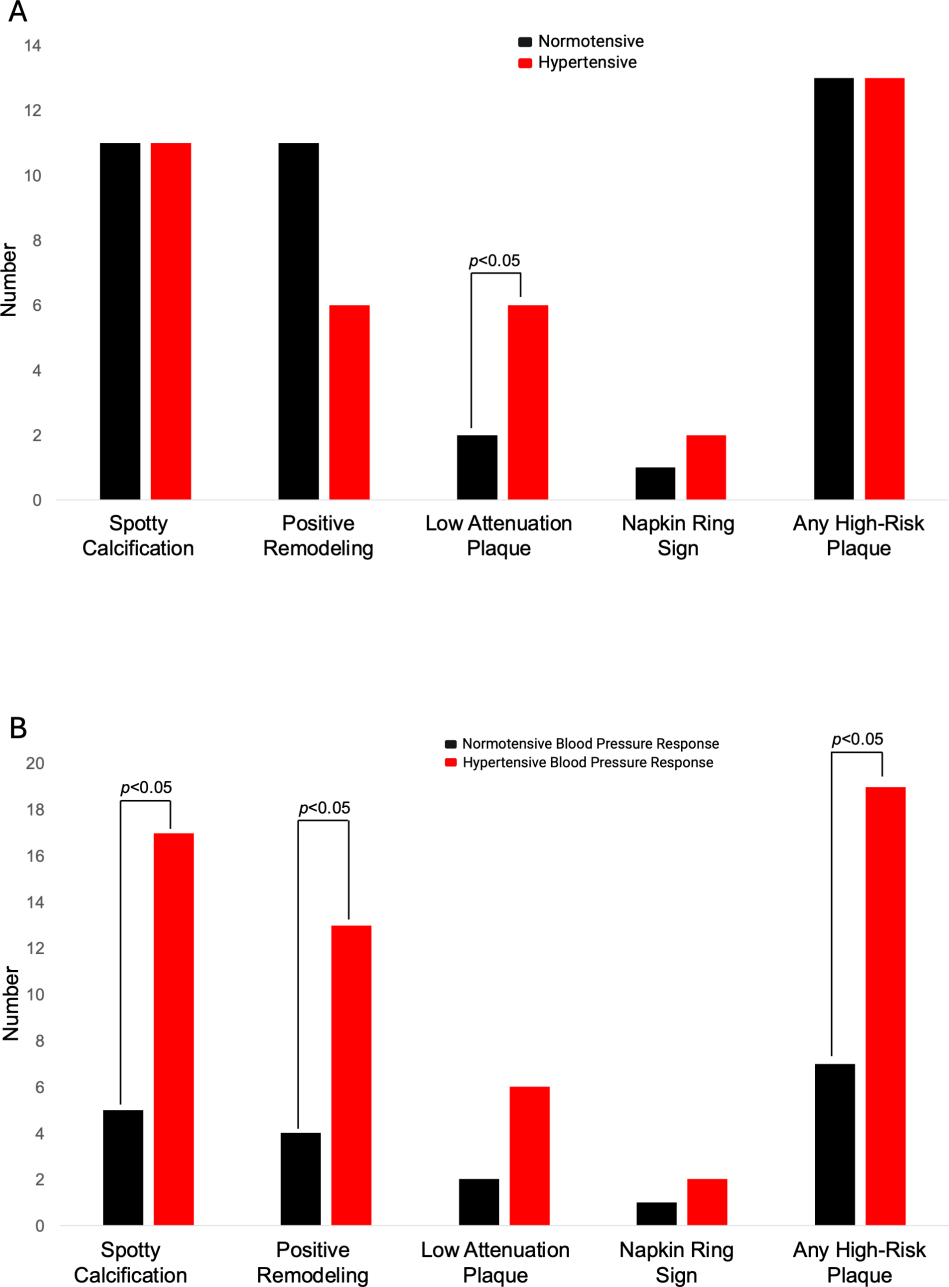
High-risk plaque features on coronary CT angiography. (**A**) Normotensive versus hypertensive athletes and (B) normotensive versus hypertensive exercise responders.

Hypertensive athletes had a significantly greater prevalence of CAC scores >100 AU, coronary plaque in two vessels, luminal stenosis >50% and low-attenuation plaque compared with normotensive athletes (figure 3A). However, there was no significant difference between the groups with respect to the coronary plaque morphology (table 2).

**Table 2:** Coronary artery features in normotensive and hypertensive athletes and in athletes with normotensive or hypertensive responses to exercise

Characteristics	Normotensive (n=120)	Hypertensive (n=78)	P value	Normotensive blood pressure response (n=105)	Hypertensive blood pressure response (n=93)	P value
CAC score (AU)	0.00 (0.00, 13.3)	1.20 (0.00, 74.5)	0.055	0.00 (0.00, 7.80)	2.00 (0.00, 59.2)	0.215
CAC score >0 AU (%)	52 (43.3)	42 (53.8)	0.148	43 (41)	51 (54.8)	0.051
CAC score >100 AU (%)	14 (11.7)	18 (23.1)	0.033	15 (14.3)	17 (18.3)	0.446
CAC score >400 AU (%)	6 (5)	9 (11.5)	0.089	7 (6.7)	8 (8.6)	0.607
CAC score >75th percentile (%)	26 (21.7)	25 (32.1)	0.103	22 (21.0)	29 (31.2)	0.100
Number of diseased vessels (%)						
0	67 (55.8)	35 (44.9)	0.132	61 (58.1)	41 (44.1)	0.049
1	36 (30)	22 (28.2)	0.640	25 (23.8)	32 (34.4)	0.100
2	8 (6.7)	12 (15.4)	0.014	12 (11.4)	10 (10.8)	0.880
3	9 (7.5)	8 (10.3)	0.499	7 (6.7)	10 (10.8)	0.306
Plaque morphology (%)						
None	67 (55.8)	35 (44.9)	0.132	61 (58.1)	41 (44.1)	0.049
Calcified	34 (28.3)	28 (35.9)	0.262	29 (27.6)	33 (35.5)	0.234
Mixed	18 (15)	14 (17.9)	0.582	14 (13.3)	18 (19.4)	0.251
Non-calcified	1 (0.8)	1 (1.3)	0.758	1 (1.0)	1 (1.1)	0.931
Coronary artery plaque site (%)						
LMS	3 (2.5)	6 (7.7)	0.087	5 (4.8)	4 (3.2)	0.877
LAD	45 (37.5)	36 (46.2)	0.226	36 (34.3)	45 (48.4)	0.044
LCx	18 (15)	16 (20.5)	0.315	17 (16.2)	17 (14.0)	0.697
RCA	16 (13.3)	18 (23.1)	0.076	16 (15.2)	18 (17.2)	0.443
Stenosis >50%	9 (7.5)	15 (19.2)	0.014	5 (4.8)	19 (20.4)	<0.001
High-risk plaque features (%)						
Spotty calcification	11 (9.2)	11 (14.1)	0.266	5 (4.8)	17 (18.3)	0.002
Positive remodelling	11 (9.2)	6 (7.7)	0.737	4 (3.8)	13 (14.0)	0.010
Low attenuation plaque	2 (1.7)	6 (7.7)	0.034	2 (1.9)	6 (6.5)	0.101
Napkin ring sign	1 (0.8)	2 (2.6)	0.324	1 (1.0)	2 (2.2)	0.485
Any high-risk plaque	13 (10.8)	13 (16.7)	0.221	7 (6.7)	19 (20.4)	0.004

AU, agatston units; CAC, coronary artery calcium; LAD, left anterior descending; LCx, left circumflex; LMS, left main stem; RCA, right coronary artery.

### Exercise performance and blood pressure response

All athletes underwent a cardiopulmonary exercise stress test on a cycle ergometer. The mean maximal power output was 331±53 Watts, which equated to 14.2±2.0 METS. The p$\dot{V}$O_2_ was 49.9±7 mL⋅kg^−1^⋅min^−1^ (154±19% age predicted aerobic capacity). The average maximum percentage heart rate achieved was 97%±7% of the maximum predicted for age. The average maximum systolic and diastolic exercise BP during CPET was 206±23 mm Hg and 78±9 mm Hg, respectively. In total, 93 (47%) athletes demonstrated a hypertensive response to exercise as defined as a systolic BP ≥210 mm Hg (figure 2D).^16^ Of these, 52 (56%) were hypertensive on 24-hour ABP monitoring.

Athletes who exhibited a hypertensive response to exercise achieved a significantly greater per cent predicted aerobic capacity, higher ABP measures, and elevated Framingham risk score (table 1). However, no significant differences were observed between groups in peak aerobic capacity, peak workload or workload adjusted for blood pressure. In addition, a hypertensive response to exercise was associated with disease of the left anterior descending (LAD) coronary artery, coronary stenosis >50%, and the high-risk plaque markers spotty calcification and positive remodelling (figure 3B). Notably, although the distribution of plaque morphology types (calcified, non-calcified and mixed) did not differ between groups, a significantly smaller proportion of hypertensive responders had no detectable coronary plaque, suggesting a higher overall burden of subclinical atherosclerosis in this group (table 2).

### Coronary calcium score and coronary plaques

Ninety-four (47%) athletes had a CAC score 1–99, 32 (16%) athletes had a CAC score 100–399 AU and 15 (8%) athletes had a CAC score ≥400 AU. Fifty-one (26%) athletes had a CAC score above the 75th percentile for the general population. Ninety-six (48%) athletes had coronary artery plaques, of which 62 (65%) were calcified, 32 (33%) were mixed and 2 (2%) were non-calcified. Most plaques were observed in the LAD artery (51%). Twenty-four (25%) athletes had a coronary stenosis ≥50%. Among these athletes, the median CAC score was 252.9 (IQR 82.7 to 818.0), with a range of 0–1930.2. Twenty-six (27%) athletes had high-risk plaque, of whom 22 (85%) had spotty calcification, 17 (65%) had positive remodelling, 8 (31%) had low attenuation plaque and 3 (12%) had napkin ring sign.

### Relationship between hypertension and CAD characteristics

In univariate analysis, hypertension was significantly associated with a CAC score ≥100 AU (OR: 2.27; 95% CI 1.06 to 4.89, p=0.036), indicating a greater burden of coronary atherosclerosis in this group (table 3). After multivariate adjustment for age, lifetime exercise dose and office blood pressure, ambulatory hypertension was independently associated with a CAC score ≥100 AU (OR: 2.56; 95% CI 1.08 to 6.04, p=0.032). Hypertension was also significantly associated with coronary stenosis >50% in both unadjusted (OR: 2.91; 95% CI 1.20 to 7.03, p=0.018) and adjusted models (OR: 2.92; 95% CI 1.17 to 7.33, p=0.022).

**Table 3:** Univariate and multivariate logistic regression analysis of the relationship of coronary artery disease measures and blood pressure

Characteristic	Hypertension				Hypertensive response to exercise			
	**Univariate** **OR (95% CI)**	**P value**	**Multivariate** **OR (95% CI)**	**P value**	**Univariate** **OR (95% CI)**	**P value**	**Multivariate** **OR (95% CI)**	**P value**
CAC score >0 AU (%)	1.53 (0.86 to 2.71)	0.134	–	–	1.75 (1.00 to 3.08)	0.052	–	–
CAC score ≥100 AU (%)	2.27 (1.06 to 4.89)	0.036	2.56 (1.08 to 6.04)	0.032	1.38 (0.65 to 2.92)	0.397	–	–
CAC score ≥400 AU (%)	2.48 (0.85 to 7.26)	0.098	–	–	1.45 (0.52 to 4.05)	0.482	–	–
Coronary stenosis >50%	2.91 (1.20 to 7.03)	0.018	2.92 (1.17 to 7.33)	0.022	5.08 (1.82 to 14.2)	0.002	4.72 (1.65 to 13.5)	0.004
Coronary plaque	1.55 (0.88 to 2.76)	0.132	–	–	1.76 (1.01 to 3.09)	0.049	1.63 (0.86 to 3.06)	0.132
High-risk plaque	1.67 (0.73 to 3.83)	0.224	–	–	3.64 (1.46 to 9.13)	0.006	3.27 (1.27 to 8.43)	0.014

Age, lifetime exercise dose and resting clinic hypertension were used as covariates in the multivariate model.

AU, agatston units; CAC, coronary artery calcium.

**Figure 4:**
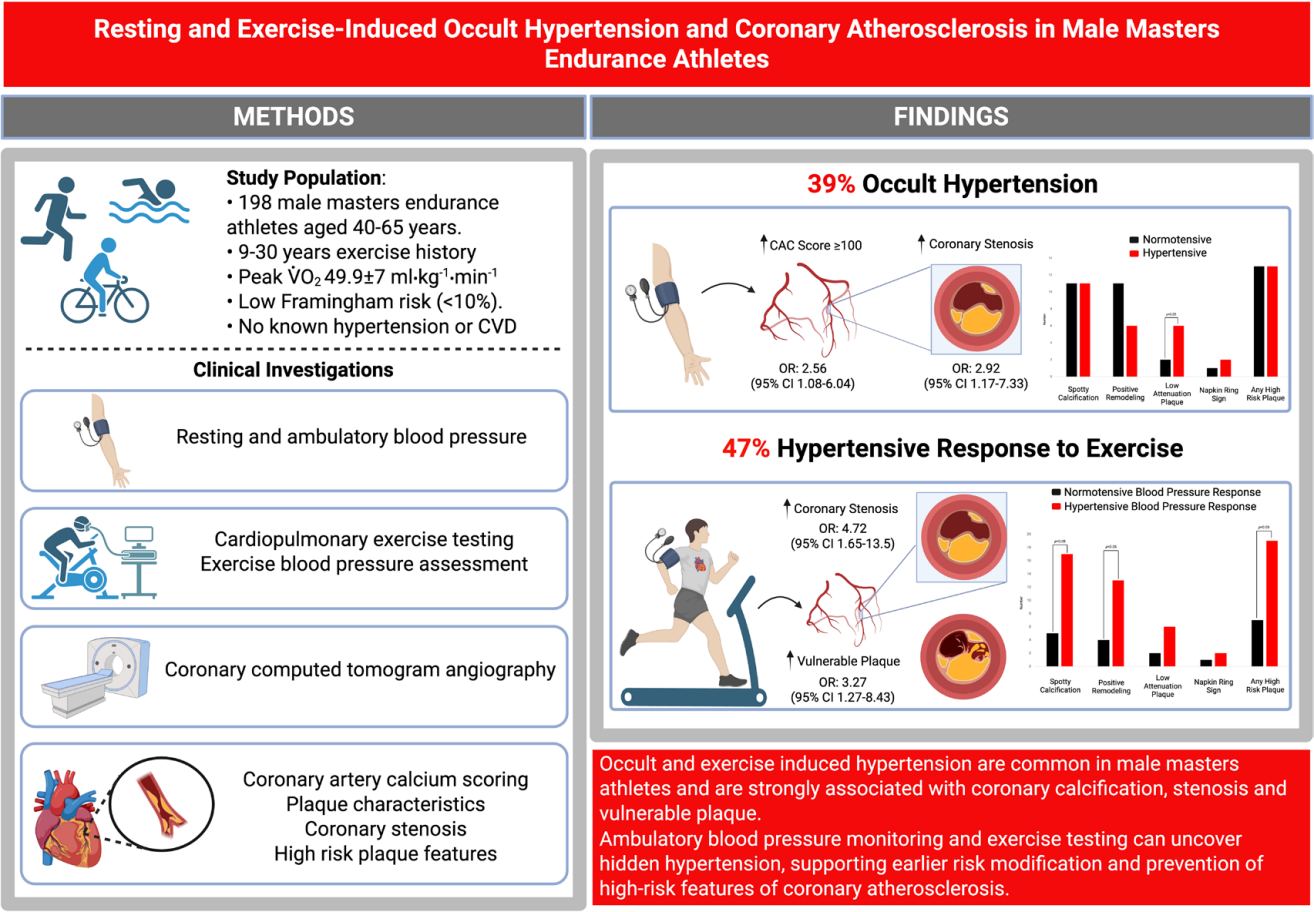
Central Illustration. Occult and exercise-induced hypertension are associated with high-risk coronary atherosclerosis in male masters endurance athletes. CAC, coronary artery calcification; CVD, cardiovascular disease. Created with BioRender.com.

In univariate analysis, a hypertensive response to exercise was significantly associated with coronary stenosis >50% (OR: 5.08; 95% CI 1.82 to 14.2, p=0.002), the presence of coronary plaque (OR: 1.76; 95% CI 1.01 to 3.09, p=0.049) and high-risk plaque features (OR: 3.64; 95% CI 1.46 to 9.13, p=0.006). Following multivariate adjustment for age, lifetime exercise dose and office blood pressure, a hypertensive response to exercise remained independently associated with coronary stenosis >50% (OR: 4.72; 95% CI 1.65 to 13.5, p=0.004), and high-risk plaque (OR: 3.27; 95% CI 1.27 to 8.43, p=0.014). However, a hypertensive response during exercise was not associated with CAC scores (>0, ≥100 or ≥400 AU, table 3). These relationships are summarised in figure 4.

### Exploratory combined phenotype analysis

In an exploratory analysis, 52 (26%) masters athletes exhibited both hypertension and a hypertensive BP response to exercise. In univariate analysis, the presence of both hypertension and a hypertensive BP response to exercise was significantly associated with coronary stenosis >50% (OR: 4.06; 95% CI 1.69 to 9.78, p=0.002) and high-risk plaque features (OR: 2.40; 95% CI 1.02 to 5.65, p=0.045). Following multivariate adjustment for age, lifetime exercise dose and resting clinic blood pressure, this combined blood pressure phenotype remained independently associated with coronary stenosis >50% (OR: 3.64; 95% CI 1.43 to 9.30, p=0.007) only. The association with high-risk plaque was directionally consistent but did not reach statistical significance.

### Clinical management

In addition to the athletes requiring further management for hypertension, 54 (25%) athletes presented with cardiac findings that warranted further investigation, management and/or follow-up. Forty-eight athletes (22%) with evidence of CAD received statins. Three (1.4%) athletes underwent percutaneous coronary intervention (PCI) with drug-eluting stents, and one (0.5%) athlete underwent coronary artery bypass grafting (CABG). All athletes undergoing revascularisation were asymptomatic, and treatment decisions were based on the presence of significant coronary disease affecting one or more vessels. Of the three athletes who underwent PCI, two had fractional flow reserve (FFR)-guided intervention, while the third proceeded directly to PCI based on severe disease on invasive angiography. The athlete undergoing CABG had invasive coronary angiography and cardiac MRI demonstrating a prior infarction in the LAD territory, with multivessel disease including an occluded LAD and severe disease in the first diagonal and obtuse marginal branches. Additionally, 3 (1.4%) athletes were identified with paroxysmal atrial fibrillation, 2 (1%) had a bicuspid aortic valve, 2 (1%) had myocarditis with left ventricular impairment and 2 (1%) had a dilated ascending aorta.

## Discussion

This study provides new insights into the association between hypertension and coronary disease in masters athletes, particularly regarding CAC, coronary stenosis and the presence of high-risk plaque. Our study results demonstrate that 39% of male masters athletes have occult hypertension according to the most recent criteria, which is associated with 2.6 times greater odds of a CAC score ≥100 AU and 2.9 times greater odds of coronary stenosis >50%, independent of age, lifetime exercise exposure and resting clinic BP. Furthermore, a hypertensive response to exercise was associated with 4.7 times greater odds of coronary stenosis >50% and 3.3 times greater odds for high-risk plaque, also independent of age, lifetime exercise dose and resting clinic hypertension. These findings highlight the risks of untreated hypertension in male masters athletes. Notably, athletes exhibiting both occult hypertension and a hypertensive BP response to exercise demonstrated a strong association with coronary stenosis >50%, although these findings should be interpreted cautiously given the exploratory nature of this analysis.

Habitual physical activity is generally considered protective against CAD; however, growing evidence suggests that male masters athletes exhibit higher CAC scores and greater prevalence of coronary atherosclerosis on CCTA compared with age and atherosclerotic risk-matched controls.^4 20^ Two studies reported that athletes with plaque were more likely to have calcified plaque, while non-athletes tended to have mixed plaques more frequently.^2 4^ These findings were interpreted as relatively benign, as calcified plaques are considered more stable and less prone to rupture compared with mixed or non-calcified plaques, which are associated with a higher risk of adverse cardiovascular events.^21 22^ However, recent findings report that lifelong endurance athletes had more coronary plaques, including more non-calcified plaques, compared with fit and healthy individuals with a similarly low cardiovascular risk profile.^23^

Although cardiovascular fitness reduces the risk of ischaemic events regardless of CAC score,^24^ there is emerging evidence that a high CAC is linked with an increased prevalence of adverse coronary events irrespective of fitness. Indeed, 4 (2%) of the masters athletes in our study required invasive coronary intervention. These findings underscore the importance of comprehensive BP monitoring in identifying and managing cardiovascular risk in this population.

### Clinical implications

Hypertension is recognised as a primary preventable cause of CAD worldwide^14^ and is predictive of the presence of CAC in the general population^25^ and athletic individuals.^11^ Although aerobic exercise training reduces systolic and diastolic BP by 4.5 mm Hg and 2.5 mm Hg, respectively,^26^ and regular moderate-intensity exercise has been shown to reduce mortality by 15%,^27^ our findings suggest that even in the context of these beneficial effects, a significant proportion of masters athletes have occult hypertension that would not be identified by resting clinic measurements alone. Importantly, when a CAC score ≥100 AU or significant CAD (coronary stenosis >50%) is identified in a masters athlete, ABP monitoring should be considered to detect occult hypertension that may be contributing to coronary atherosclerosis and may be amenable to treatment. In the present cohort, nearly 90% of athletes diagnosed with hypertension on ambulatory monitoring had a normal resting clinic BP, underscoring the clinical value of ambulatory assessment in this population.

Notably, a hypertensive BP response to exercise was predictive of coronary artery stenosis >50% and the presence of high-risk plaque. The modest overlap between ambulatory and exercise-induced hypertension suggests that these assessments identify distinct blood pressure phenotypes rather than representing interchangeable measures of the same condition. Accordingly, exercise testing should not be used to exclude occult hypertension, but may provide complementary information regarding cardiovascular risk, particularly in identifying athletes with a propensity towards obstructive disease and high-risk plaque. A prior smaller study of 50 normotensive master athletes revealed that 28 exhibited a hypertensive BP response. These athletes demonstrated a high prevalence of CAC ≥10 or ≥100 AU, a greater number of coronary plaques, a higher number of vessels affected, and a higher prevalence of luminal reduction by plaques compared with athletes with a normal BP response.^28^ A hypertensive BP response during exercise is an independent predictor of hypertension, regardless of resting BP levels in the general population^29^ and among athletes.^30^ Identification and safety netting of such athletes with advice regarding BP monitoring and control could mitigate progression of atherosclerosis, development of high-risk plaques and the risk of adverse events. However, given the high prevalence of a hypertensive BP response when defined using a fixed systolic threshold,^31^ non-invasive maximal exercise testing should not be interpreted as a diagnostic test for hypertension in isolation. Rather, exercise BP responses should be interpreted in the context of workload achieved, overall cardiovascular risk, and adjunctive assessments such as ABP monitoring.

Based on our observations, recommendations for the cardiovascular evaluation of masters athletes should include a low threshold for ABP monitoring, particularly in those with evidence of CAC or CAD, as a formal part of their clinical evaluation and risk stratification, to improve detection of occult hypertension in this cohort. Non-invasive maximal exercise testing should be considered selectively, particularly in athletes with elevated CAC scores, evidence of coronary plaque or stenosis on imaging, an exaggerated BP response during exercise, or a higher overall cardiovascular risk profile. These investigations are both widely available (in some settings), low-cost and non-invasive. Consideration should be given to amending guidance to reflect that CCTA may enhance risk stratification in this population since direct markers of coronary risk, including plaque morphology and plaque vulnerability markers, are known to be associated with increased coronary events.^32 33^ This modification in approach can also be justified, given the rapid advancement in CT technology, resulting in ever-reducing radiation doses associated with CCTA and with new techniques such as FFR, enabling CCTA to offer both structural and functional evaluation.

### Strengths and limitations

The cohort is representative of ostensibly healthy, asymptomatic male recreational endurance athletes aged 40–65 years, who regularly engage in moderate to intense exercise (median 9.3 hours per week; 7–12 hour), for many years (median 16 years; 9–30 years). The athletes maintain a high level of fitness with a mean p$\dot{V}$O_2_ of 49.9±7 mL⋅kg^−1^⋅min^−1^ (154%±19% age-predicted aerobic capacity). However, our study does have limitations. This study did not include matched controls; therefore, it was not possible to easily compare this data to non-athletes. However, the objective was to examine the relationship of CAD risk markers with BP parameters, a relationship which is well described in the literature among the non-athletes. Additionally, this study only examined men; therefore, the data cannot be reliably extrapolated to their female counterparts. Our existing experience of masters female athletes indicates a low prevalence of coronary atherosclerosis with a more favourable profile compared with healthy non-athletes of similar age.^34^ Large data from other studies have also reported a lack of association between reported physical activity, CAC, and adverse coronary events in exercising females, including endurance athletes.^6 35^ Thirdly, most athletes were of Caucasian ethnicity, and previous data suggests variations in CAC scores among ethnic groups, again necessitating caution in applying this data in the context of other ethnic groups.

Although BP is a significant contributor to CAD in this cohort, additional undetermined factors warrant further research. This study did not explore or account for the role of diet, alcohol consumption, substance use, lipoprotein(a), inflammatory biomarkers or polygenic risk for CAD, which are considered relevant in the pathophysiology of both hypertension and CAD in the general population. Furthermore, although peak systolic BP during exercise was indexed to achieved workload and did not differ between groups, we did not assess the systolic blood pressure–workload slope across exercise stages. Such analyses may better account for fitness-related differences in cardiac output and vascular responses during graded exercise testing, as fixed systolic blood pressure thresholds may overestimate the prevalence of a hypertensive response in highly trained endurance athletes.

## Conclusion

Masters male endurance athletes have a high prevalence of occult hypertension, which is independently associated with high CAC scores, coronary stenosis and high-risk plaques. These findings suggest that blood pressure should be actively monitored and managed even in highly active individuals, as male masters endurance athletes are not protected from hypertension-related coronary risk. A comprehensive cardiovascular evaluation, including selective non-invasive maximal exercise testing, ABP monitoring, and consideration of CCTA, may improve risk stratification and targeted management in this population.

## Data Availability

Data are available upon reasonable request.
